# Differential Production of Phenolics, Lipids, Carbohydrates and Proteins in Stressed and Unstressed Aquatic Plants, *Azolla filiculoides* and *Azolla pinnata*

**DOI:** 10.3390/biology9100342

**Published:** 2020-10-19

**Authors:** Thi Linh Nham Tran, Ana F. Miranda, Shamila Weerakoon Abeynayake, Aidyn Mouradov

**Affiliations:** 1 School of Sciences, RMIT University, Bundoora, VIC 3083, Australia; s3654084@student.rmit.edu.au (T.L.N.T.); ana.miranda@rmit.edu.au (A.F.M.); s.abeynayake@latrobe.edu.au (S.W.A.); 2 Faculty of Agriculture, Bac Lieu University, 8 wards, Bac Lieu 960000, Vietnam; 3 Department of Animal, Plant and Soil Sciences, Centre for AgriBiosciences, La Trobe University, Bundoora, VIC 3086, Australia

**Keywords:** anthocyanins, condensed tannins, fatty acid methyl esters (FAMEs), flavonoids, nutrients

## Abstract

**Simple Summary:**

Crops are the primary feedstock for the production of food/feed and energy, and our dependency on them has been increasing over the last decade. Climate change, reduced land for active agriculture, increased population, reduced amount of clean water, and high costs of chemical fertilizers and energy make modern agriculture unsustainable. Demands are rising for the next generation of cheap and sustainable feedstock, which could be productive under existing conditions. Aquatic plants, such as the free-floating species duckweed and Azolla, have started attracting attention because of their unique features, presenting advantages over terrestrial plants. Terrestrial plants grow on the land or need to be on dry land to survive. On the other hand, aquatic plants have the ability to thrive in ponds under controlled conditions using wastewaters as a source of nutrients. Over the last decade, Azolla species became an attractive feedstock for livestock because of their accumulation of valuable products, phenolic compounds, proteins, lipids, and carbohydrates. Stress-triggered changes in these nutritional components could have a significant impact on the nutritional value of Azolla, which is used as a sustainable food supplement for livestock, poultry, and fish industries. This paper aims to assess the effects of environmental and nutrient stresses on the biosynthesis of valuable metabolites in Azolla.

**Abstract:**

The metabolic plasticity of shikimate and phenylpropanoid pathways redirects carbon flow to different sink products in order to protect sessile plants from environmental stresses. This study assessed the biochemical responses of two Azolla species, *A. filiculoides* and *A. pinnata*, to the combined effects of environmental and nutritional stresses experienced while growing outdoors under Australian summer conditions. These stresses triggered a more than 2-fold increase in the production of total phenols and their representatives, anthocyanins (up to 18-fold), flavonoids (up to 4.7-fold), and condensed tannins (up to 2.7-fold), which led to intense red coloration of the leaves. These changes were also associated with an increase in the concentration of carbohydrates and a decrease in concentrations of lipids and total proteins. Changes in lipid biosynthesis did not cause significant changes in concentrations of palmitoleic acid (C16:0), linolenic acid (C18:3), and linoleic acid (C18:2), the fatty acid signatures of Azolla species. However, a reduction in protein production triggered changes in biosynthesis of alanine, arginine, leucine, tyrosine, threonine, valine, and methionine amino acids. Stress-triggered changes in key nutritional components, phenolics, lipids, proteins, and carbohydrates could have a significant impact on the nutritional value of both Azolla species, which are widely used as a sustainable food supplement for livestock, poultry, and fish industries.

## 1. Introduction

Crops are the primary feedstock for the production of food/feed and energy, and our dependency on them has increased over the last decade. Climate change, reduced land for active agriculture, increased population, reduced amount of clean water, along with high costs of chemical fertilizers and energy, make modern agriculture unsustainable [1,2]. Demands are rising for the next generation of cheap and sustainable feedstock which could be productive under existing conditions. Microalgae tick all the boxes for producing a broad spectrum of nutrients and showing the capacity to grow rapidly on wastewater nutrients [3,4,5]. However, challenges with harvesting under large scale production make their technology expensive [6,7]. Aquatic plants, such as the free-floating species duckweed and Azolla, have started attracting attention because of their unique features, which present the advantages of microalgae with easy and cheap harvesting technologies [8,9,10,11,12,13]. Azolla species grow in a symbiotic association with *Nostoc* (*Anabaena) azollae*, a nitrogen-fixing cyanobacterium that allows its host plant to grow rapidly in nitrogen-free solutions [14,15,16]. The application of this species for wastewater treatment and the production of biomass for food/feed and as feedstock for bioenergy has been broadly investigated [9,13,17,18,19,20]. Azolla thrives in tropical to temperate regions of the world [15,21]. When grown under optimal conditions, this species can double its biomass every two to four days, with average annual productivity of 39 t/hectare [11]. Over the last decade, Azolla species became an attractive feedstock for livestock because of the accumulation of valuable products, such as proteins (up to 400 g/kg DW), lipids (160 g/kg DW), and phenolics comprising antioxidants such as flavonoids, tannins, and anthocyanins (50 g/kg DW in total) [11,13,20,22,23,24].

Fresh Azolla can replace up to 50% of feed concentrate for cows, decreasing feed and labor costs by 16.5% and the cost of milk production by 18.5% [25]. The addition of fresh Azolla to the diet of native chickens, laying hens, and grazing ducks has also been shown to reduce the feeding costs significantly without affecting egg weight and other quality parameters [26]. The use of dried Azolla at 7.5% resulted in a 2.6% increase in the bodyweight of broilers and contributed to a better immune response [27]. Moreover, Azolla has been widely used as a low-cost and a high nutritional supplement in fish meal [28].

Azolla’s protein level and composition of amino acids are the main nutritional components that make it a valuable feed supplement [11,29,30]. An average yield of dry Azolla biomass of 35 t DW/ha-year correlates with an annual protein production of 7–9 t DW/ha-year [29]. This is up to 3-fold higher than the soybean, which yields up to 2 t protein/ha-year [11,31]. An additional advantage of Azolla-based protein production is related to the fact that under favorable conditions these species can be grown and collected throughout the year, whereas the production of soybean grains is seasonal (once per year). Azolla can grow without nitrogen due to its nitrogen-fixing capacity, while commercial-scale soybean production still requires at least 150 kg of N fertilizer ha^−1^ per year [11,32]. However, Azolla has a lower protein content compared to Spirulina, a cyanobacterium commonly used as a food source. Spirulina (*Arthrospira* sp.) contains around 48.9% protein and can produce in semi-continuous cultures up to 25 t protein/ha-year [33]. However, harvesting and concentration of cyanobacteria and algae still account for 30–40% of the product’s cost [7].

Phenolic compounds, the products of shikimate and phenylpropanoids pathways, comprise more than 8000 compounds, including flavonoids, flavonols, flavones, anthocyanins, tannins and condensed tannins (proanthocyanins), and other benzene (C6) ring-containing molecules [34,35]. The most abundant phenolic compounds identified in Azolla include anthocyanidins, coumarins, flavonols, flavones, and condensed tannins [23,35]. The antioxidation mechanism triggered by plant phenolics has been shown to benefit human health, including a reduced risk of developing cardiovascular diseases, cancer, diabetes type 2, and neurological diseases [36,37,38]. Condensed tannins are the most abundant phenolic component extracted from Azolla species [10,17]. Because of their health benefits, which include anticancer, antidiabetic, neuroprotective, and antimicrobial properties, they became popular as a food/feed supplement [39]. As livestock feed, low concentrations of these polymers can non-specifically bind food proteins, reducing their fermentation in gastrointestinal tracts and reducing ruminal bloats [17]. A high concentration of phenols and condensed tannins, however, can have a negative impact on the nutritional value of Azolla plants, reducing their digestibility [11,29,30,40,41,42]. Phenolics from Azolla have been extensively characterized previously [10,35,43].

The metabolic plasticity of shikimate and phenylpropanoid pathways redirecting carbon flow to the biosynthesis of different phenolic compounds and other carbon sinks, such as carbohydrates, lipids, and proteins, is a crucial evolutional factor developed by sessile plants to protect them against biotic and abiotic stresses [44,45,46]. Stressed Azolla plants show the intense red coloration of their leaves, indicating enhanced production of colored phenolic pigments (Figure S1). Earlier, we showed that this triggered differential accumulation of metabolites from the phenylpropanoid and fatty acid biosynthesis pathways when plants were grown indoors, in glasshouse conditions [20,47]. In this study, we assessed the biochemical responses of two Azolla species, *A. filiculoides* and *A. pinnata*, to nutrient and environmental stresses experienced while growing outdoors under summer conditions in Australia. These two species differ in habitat, growth rates, and genetic makeup (i.e., *A. filiculoides* grows in temperate climates in Europe and *A. pinnata* grows in tropical climates in Asia and Australia) [48,49]. To our knowledge, this is the first comprehensive study of biochemical responses of two Azolla species, showing differential accumulation of the key nutrients (anthocyanins, flavonoids, condensed tannins, carbohydrates, lipids, and proteins) in response to stressed conditions.

## 2. Materials and Methods

### 2.1. Growth Conditions

Two aquatic plants, *A. filiculoides* and *A. pinnata,* were obtained from the RMIT collection of aquatic plants and algal species. As a stock, both plants were grown in 250-mL plastic boxes (11 cm × 18 cm) in half strength (1/2SH) Schenk and Hildebrandt’s medium [49] in a glasshouse with a 16-h photoperiod, 25 °C, and a photosynthetic photon flux density of 100 μm/m^2^-s provided by fluorescent tubes. In further experiments, *A. filiculoides* and *A. pinnata* plants were grown in 1.3 m (length) × 0.7 m (width) × 0.50 m (depth) tanks in 1/2SH medium without nitrogen. Plants were stressed by starving and growing under direct exposure to sunlight during Australian summer conditions (light intensity 5–10 Klux daytime). In control experiments, plants were regularly fed and grown under 70% shade cloth conditions, reducing light intensity to 0.5 Klux.

### 2.2. Biochemical Analysis

#### 2.2.1. Lipid Extraction of Fatty Acid Methyl Esters

Lipid extraction and fatty acid methyl esters (FAMEs) analysis was conducted according to Miranda et al. [20], with minor modifications. Briefly, 5 mL of solvent (chloroform/methanol 2:1) was added to a tube containing 50 mg of ground Azolla and was left to shake at 200 rpm overnight. Samples were then vortexed at the maximum speed for 1 min and centrifuged at 3000 rpm for 30 min. The resulting supernatant was transferred to a new tube, and 2 mL of NaCl was added before vortexing for 5 s. After that, the tubes were centrifuged at 3000 rpm for 3 min. The lower layer was transferred into the pre-weighed tube and dried under nitrogen flow at 70 °C.

#### 2.2.2. Total Carbohydrates

Total carbohydrates were measured according to Chandran et al. [12], with minor modifications. Briefly, 2.5 mL of 2.5 N hydrochloric acid was added to 50 mg of sample. The sample was then hydrolyzed by boiling in a water bath for three hours. After cooling down, solid sodium carbonate was added to neutralize the reaction, and distilled water was added up to 50 mL. Samples were then centrifuged at 3000× *g* for 10 min at room temperature. The supernatant was then collected and 500 µL was added to a test tube. One milliliter of Milli-Q and 4 mL of anthrone reagent was added to each test tube, including the blank. After mixing well, the samples were kept in a boiling water bath for 8 min. After cooling, the absorbance of a blue-green solution was measured at 630 nm using a spectrophotometer (POLARstar Omega, BMG Labtech, Offenburg, Germany) and compared with a standard curve prepared with known amounts of glucose. The amount of total carbohydrates in the sample was calculated and expressed as g glucose equivalents/100 g sample.

#### 2.2.3. Total Protein

The total protein content was measured using the Kjeldahl method by using Foss™ Kjeldahl Digestion Systems. The nitrogen content of the samples was measured and converted to protein content by multiplying by 6.25, 4.59, and 5.0 [50].

#### 2.2.4. Total Phenolics

Total phenolic concentration was estimated using the Folin–Ciocalteu method, according to Makkar [51], with minor modifications. To 1 g of powdered sample, 7 mL of extract buffer (80% methanol) was added. The mixture was kept shaking for 12 h at 4 °C in the dark before being centrifuged at 3000× *g* for 10 min at room temperature. The supernatant was collected, and 0.5 mL of Folin–Ciocalteu reagent (1 N) was added to each sample, including a blank. After mixing using a vortex, the samples were rested for 5 min, and 2.5 mL of 5% sodium carbonate was added to all tubes, except the blank. Finally, the samples were shaken and incubated for 40 min at room temperature. The absorbance of the blue color developed against the reagent blank at 725 nm was determined using a spectrophotometer (POLARstar Omega, BMG Labtech, Offenburg, Germany).

#### 2.2.5. Total Flavonoids

The total amount of flavonoid was estimated according to Zhishen et al. [52] by using the aluminum chloride method. Briefly, 500 µL of extract (supernatant in 2.4) was diluted to 1 mL with Milli-Q water, and 150 µL of 5% sodium nitrite was added to all samples, including a blank. The tubes were then mixed and incubated at room temperature for 5 min before adding 150 µL of 10% aluminum chloride to all the test tubes, including the blank. The mixture was mixed and further incubated for 6 min before adding 2 mL of 4% sodium hydroxide to all the test tubes. The contents of the tubes were made up to 5 mL using Milli-Q water, and the contents were mixed well using a vortex. The samples were rested for 15 min at room temperature, and the absorbance of the pink color that had developed was measured against the blank at 510 nm using a spectrophotometer (POLARstar Omega, BMG. Labtech, Offenburg, Germany) and estimated based on the amounts of rutin as standard and expressed as mg/standard equivalent per gram dry powder for the samples.

#### 2.2.6. Total Condensed Tannins

The amount of total condensed tannins was measured as described by Ray et al. [53], with minor modifications. To each gram of sample powder, 3 mL of extract buffer (80% methanol and 5.3 mM sodium bisulfite) was added in the dark. The mixture was kept shaking for 12 h at room temperature and centrifuged at 3000× *g* for 10 min at room temperature. The supernatant containing the methanol-soluble fractions was collected and analyzed according to Ray et al. [53] and using catechin as standard.

#### 2.2.7. DMACA Staining of Condensed Tannins

The stain 4-dimethylaminocinnamaldehyde (DMACA) was used to detect condensed tannins in aquatic plants using the method described in Abeynayake et al. [54]. Briefly, plants were decolorized in absolute ethanol for 3 h and stained for the presence of proanthocyanidins and flavan-3-ols using 0.01% (*w*/*v*) 4-dimethylaminocinnamaldehyde (DMACA.) in absolute ethanol containing 0.8% *w*/*v* hydrochloric acid for 2 h [55]. After this, plants were transferred to 100% ethanol and further vacuum-infiltrated for 1 min with fixative (6% *w*/*v* glutaraldehyde, 4% *w*/*v* paraformaldehyde in 50 mM sodium phosphate buffer, pH 7.4) before being incubated for 2 h at 4 °C. Finally, plants were washed three times (5 min) in 50 mM sodium phosphate buffer, pH 7.4. For making sections, tissues were embedded in LR White or paraffin matrices, followed by DMACA staining [55]. Transverse and longitudinal sections of embedded samples in LR White resin (6–10 μm) and paraffin (8 μm) were generated using a microtome. Cell walls were stained with 0.05% toluidine blue (ProSciTech, Kirwan, QLD, Australia, Cat. # C078). Images were captured using a Leica MZFLIII light microscope (Leica, Wetzlar, Germany).

#### 2.2.8. Anthocyanin Levels

Anthocyanin levels were measured as in Neff et al. [56], with minor modifications. To 1 g of powdered sample, 3 mL of methanol 1% HCl was added in the dark. The mixture was shaken overnight in a dark room at 4 °C. After that, 2 mL of Milli-Q water and 5 mL of chloroform was added to the samples. The samples were centrifuged at 8000× *g* for 5 min at 4 °C. The supernatant was collected, and anthocyanin levels were estimated following [56] and by measuring the absorbance at 530 and 657 nm using a spectrophotometer (POLARstar Omega, BMG Labtech, Offenburg, Germany). The relative amount of anthocyanin was calculated by subtracting the A657 from the A530.

### 2.3. Statistical Analysis

All experiments in this study were conducted in triplicate. All data are expressed as a mean ± standard deviation. The experimental data were subjected to the one-way analysis of variance (ANOVA) as implemented in the GraphPad InStat 3 statistics platform, San Diego, CA, USA. Tukey simultaneous tests were conducted to determine the statistical differences between treatments. To ascertain that the observed variations were statistically significant, the probability (P) values were determined. A 95% confidence level (*p* < 0.05) was applied for all analyses.

## 3. Results and Discussion

### 3.1. Accumulation of Phenolic Compounds in Green and Red A. filiculoides and A. pinnata Plants

**Figure 1:**
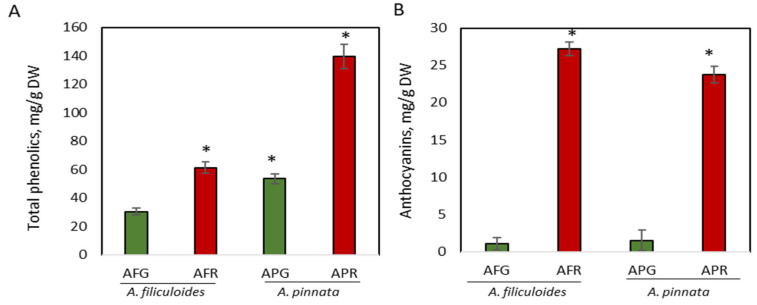
Accumulation of total phenolics (**A**) and anthocyanins (**B**) in green and red *A. filiculoides* and *A. pinnata.* AFG: *A. filiculoides*_Green; AFR: *A. filiculoides*_Red; APG: *A. pinnata*_Green; APR: *A. pinnata*_Red. (ANOVA; * *p* < 0.05 compared to AFG).

*A. filiculoides* and *A. pinnata* plants growing outdoors under normal and stressed conditions (Material and Methods) were assessed for the concentration of phenolic compounds, total phenols, anthocyanins, flavonoids, and condensed tannins, which have a complex effect on the nutritional value of these plants [10,11]. In unstressed plants, the concentration of total phenols in *A. pinnata* was 1.7-fold higher than in *A. filiculoides*, producing 53.5 mg/g DW (5.1% DW) compared to 30.4 mg/g DW (3.1% DW) in *A. filiculoides* (*p* < 0.05) (Figure 1A). Stress increased the accumulation of phenols up to 2.6-fold in *A. pinnata*, and 2-fold in *A. filiculoides*, producing 139.5 mg/g DW (14.2% DW) and 61.4 mg/g DW (6.0% DW), respectively (Figure 1A). A higher concentration of phenolics in *A. pinnata* than in *A. filiculoides* was also shown for the same species by Brouwer et al. [11]. The enhancement of total phenolic compounds exposed to different abiotic stresses has also been shown for other Azolla species [57,58,59,60]. Unstressed *A. filiculoides* and *A. pinnata* showed similar very low concentrations of anthocyanins (Figure 1B). Stress significantly increased the concentration of anthocyanins in red *A. filiculoides* by up to 18-fold, with the concentration rising from 1.5 mg/g DW to 27.18 mg/g DW (Figure 1B). In red *A. pinnata,* the concentrations of anthocyanins showed a 15-fold increase (up to 23.8 mg/g DW) when compared to green plants. In terrestrial plants, an increased concentration of anthocyanins in response to biotic/abiotic stress conditions, such as high light intensity, cold, and pathogen infections, in order to protect from oxidative damage is a well-investigated phenomenon [61,62]. A much lower increase in the concentration of anthocyanins was observed in *Azolla imbricata* after nine days of exposure to 0.05–0.1 mg/L of cadmium [60].

It was shown that anthocyanins in frond extracts of Azolla are mainly represented by 3-deoxyanthocyanins and their acetylated glycosides, such as luteolinidin (*m*/*z* 475) and apigeninidin (*m*/*z* 459) [63,64]. High levels of deoxyanthocyanins in Azolla fronds negatively correlated with their palatability for adult *Lymnaea swinhoei* snails and *Polypedates leucomystax* tadpoles [65].

**Figure 2:**
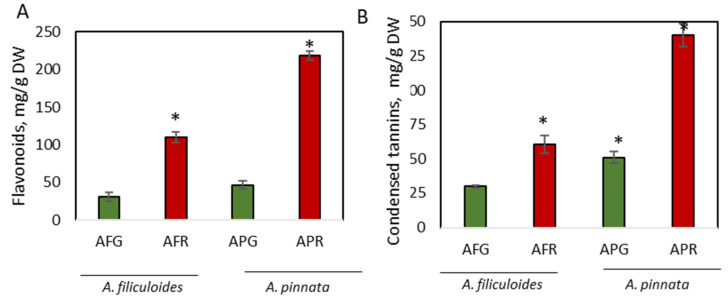
Accumulation of flavonoids (**A**) and condensed tannins (**B**) in green and red *A. filiculoides* and *A. pinnata*. AFG: *A. filiculoides*_Green; AFR: *A. filiculoides*_Red; APG: *A. pinnata*_Green; APR: *A. pinnata*_Red. (ANOVA; * *p* < 0.05 compared to AFG).

The concentration of another representative of phenolic compounds, flavonoids, were increased 3.6-fold and 4.7-fold, reaching concentrations of 109 mg/g DW and 218 mg/g DW, respectively, in stressed *A. filiculoides* and *A. pinnata* plants (Figure 2A). Both stressed and green *A. pinnata* plants contained a higher concentration of flavonoids than *A. filiculoides*. Earlier, we showed a detailed composition of flavonoids in Azolla grown on swine wastewater during the summer and winter in Australia [47]. An extensive list of the flavonoids identified in *A. filiculoides* and *A. pinnata* was previously shown [17,66]. Similarly to Azolla, the increase in the concentrations of flavonoid compounds was triggered in duckweed (*Landoltia punctata*) by nutrient starvation [67]. In terrestrial plants, the composition of flavonoids and their metabolic plasticity is mostly dependent on the plant species, their developmental stage, and environmental conditions, including both biotic and abiotic stresses [35].

**Figure 3:**
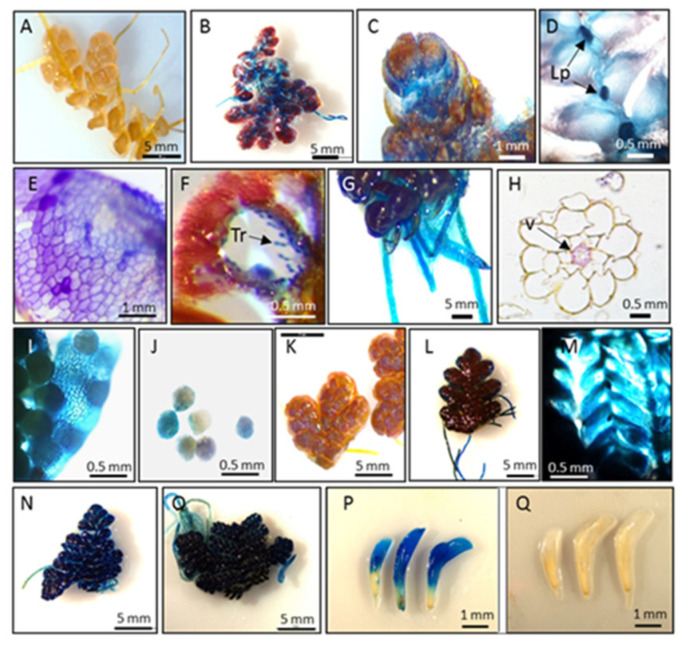
Staining of condensed tannins in *A. filiculoides* and *A. pinnata* with 4-dimethylaminocinnamaldehyde (DMACA). *A. filiculoides* green, non-stained (**A**) and stained by DMACA (**B**–**J**); leaf (**E**); leaf cavity (**F**); roots (**G**–**H**); microsporocarps (**I**) microspores (**J**); *A. filiculoides* red, non-stained (**K**) and stained with DMACA (**L**,**M**); *L. pinnata* green, stained (**N**); *L. pinnata* red, stained (**O**); white clover flowers stained with DMACA ((**P**), positive control); white clover flowers not stained with DMACA ((**Q**), negative control). Lp: leaf primordia; Tr: trichomes; V: vasculature cells.

Condensed tannins were identified as the most abundant soluble phenolic compounds in both Azolla species, accounting for 30.2 mg/g DW (3.1% DW) and 51.1 mg/g DW (5.2% DW) of the biomasses of *A. filiculoides* and *A. pinnata*, respectively (Figure 2B). Their concentrations were further increased, up to 2.7-fold, in response to stress (Figure 2A). Staining with DMCA showed a blue coloration ubiquitously accumulating in vegetative and reproductive organs (Figure 3). In leaves, blue staining was observed mainly in dorsal compared to ventral lobes (Figure 3B,C; Figure S2). Intense blue staining was detected in leaf primordia (Figure 3D). At the cellular level, blue staining was mainly observed in cell walls (Figure 3E). Within leaf cavities, strong staining was detected in trichomes (Figure 3F). Accumulation of condensed tannins in trichomes was also shown by Pereira et al. [68]. In Azolla roots, DMACA staining was detected in the vascular cells around the xylem (Figure 3G,H). The accumulation of insoluble (poly)phenols around the vascular tissue of *A. filiculoides* was previously observed, using confocal imaging, in stems and leaves *of A. filiculoides* stained with propidium periodide [17]. In reproductive organs, a well-detectable level of condensed tannins was observed in microsporocarps and microspores (Figure 3I,J). In both species, stronger coloration was detected in stressed plants compared to green plants. White clover flowers were used as a positive control for DMACA activity, since they have a high level of condensed tannins [69]. Accumulation of condensed tannins in root vasculature cells resembles accumulation profiles of lignin in the roots of terrestrial plants. Existing data describing lignin content in Azolla species is controversial, with a broad range of concentrations observed at different developmental stages, from no lignin until 41% in mature plants [23,42,70,71].

Based on the molecular analysis of flash pyrolysis and thermally assisted hydrolysis and methylation, the production of lignin was not predicted in *Azolla caroliniana* [23]. However, it is very likely that these strategies were not sensitive enough, since low levels of G-lignin-specific pyrolysis products, such as 4-(1-propenyl)guaiacol, cis-4-(2-propenyl)guaiacol, and trans-4-(2-propenyl)guaiacol, were identified in *A. filiculoides* [17]. Recent studies indicate that high concentrations of phenolic compounds, caffeoylquinic acids, and condensed tannins are responsible for reducing the nutritional value and digestibility of Azolla species [10].

However, low concentrations of condensed tannins (<3% DW) in a common forage plant, white clover, showed a beneficial effect on animal health, preventing pasture bloat and increasing nutrient uptake in ruminant livestock as a result of protection of proteins from fermentation [35,72]. Inconsistent data reflecting animal responses to the concentration of condensed tannins in the diet also correlates with their structures [73]. In terrestrial plants, the accumulation of protein-bound or fiber-bound condensed tannins protects them from vertebrates and insects [11,74]. Condensed tannins are built by two main monomers: 2,3-trans-flavan 3-ols (afzelechin, catechin, and gallocatechin) and 2,3-cis-flavan 3-ols (epiafzelechin, epicatechin, and epigallocatechin) [10,69].

### 3.2. Accumulation of Proteins, Lipids, and Carbohydrates in Green and Red A. filiculoides and A. pinnata

**Figure 4:**
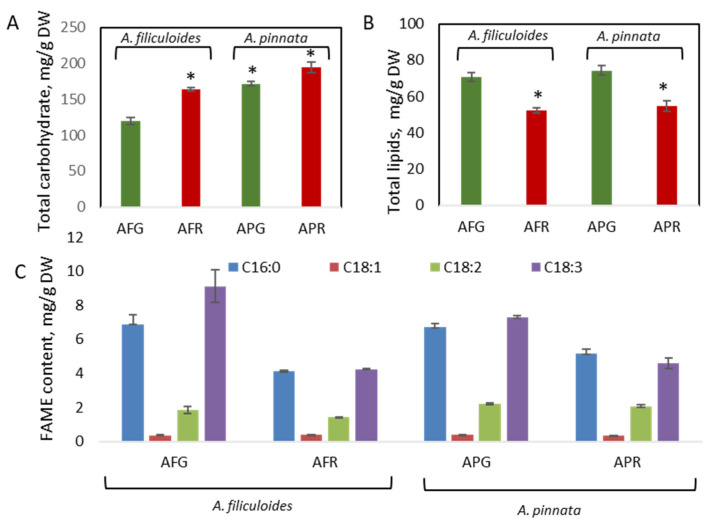
Accumulation of carbohydrates (**A**) and total lipids (**B**) in green and red *A. filiculoides* and *A. pinnata.* Fatty acid methyl ester (FAME) composition of lipids (**C**). AFG: *A. filiculoides*_Green; AFR: *A. filiculoides*_Red; APG: *A. pinnata*_Green; APR: *A. pinnata*_Red. (ANOVA; * *p* < 0.05 compared to AFG).

Green *A. pinnata* showed a significantly higher content of total carbohydrates than *A. filiculoides,* with 171 mg/g DW and 119 mg/g DW (12% DW and 17% DW), respectively (Figure 4A). Stress conditions increased the concentration of carbohydrates in both plants, with up to 164 mg/g DW and 195 mg/g DW (16% DW and 19% DW) in *A. filiculoides* and *A. pinnata*, respectively. These levels are similar to the sum of polysaccharides (19.8% DW) and soluble sugars (2.1% DW) but lower than the levels of carbohydrates observed for *A. filiculoides* (40% DW) collected from a lake in Iran [17,18].

The lipid content in green plants was around 7% DW (72 mg/g DW) and was significantly increased when plants were stressed (5% DW, 52 mg/g DW) (Figure 4B). High concentrations of palmitoleic acid (C16:0), linolenic acid (C18:3), and linoleic acid (C18:2) with lower levels of oleic acid (18:1) are FAME signatures for Azolla species (Figure 4C) [20]. In both Azolla species, stress conditions have not been shown to contribute to significant changes in the FAME profiles. Earlier, it was shown that, based on its FAME composition, Azolla could be a high-quality feedstock for biodiesel if an additional fractionation step could be used to decrease the cold filter plugging point [11,20].

Total nitrogen, protein, and amino acid contents of *A. filiculoides* and *A. pinnata* are shown in Table 1 and Table 2. Using the Kjeldahl analysis, stressed *A. filiculoides* and *A. pinnata* showed 1.4- and 1.6-fold lower concentrations of nitrogen than green plants, respectively. Table 1 shows total protein concentrations based on three known nitrogen-to-protein conversion factors: a standard nitrogen-to-protein conversion factor (6.25) based on albumin protein, as well as updated conversion factors, 4.59 and 5.0, used for marine and aquatic plants respectively [11,75,76].

Based on our results, the sum of amino acids in green *A. filiculoides* and *A. pinnata* ranged from 205 to 265 mg/g DW, which is lower than the total protein value based on the 6.25 conversion factor, but higher than for conversion factors 4.59 and 5.0 and can be averaged as 5.3. Stressed plants showed lower protein concentrations than green plants, with 141 mg/g DW and 134 mg/g DW for *A. filiculoides* and *A. pinnata*, respectively, reflecting the differences in concentrations of total nitrogen. The fact that the 6.25 conversion factor is not accurate for nitrogen-fixing plants can be explained by the accumulation of nitrogen in the form of different nitrogen-containing compounds [11]. Conversion levels for nitrogen-to-protein lower than 6.25 were observed for other aquatic plants such as duckweed (4.8), water hyacinth (4.59), and *Ulva lactuca* (4.59) [11]. The FAME composition of Azolla plants is shown in Table 2. Stress led to changes in the profiles of some amino acids in both plants. In *A. filiculoides*, it caused an increase in levels of alanine, arginine, leucine, tyrosine, threonine, and valine (*p* ≤ 0.05), while the percentage of glycine was decreased. In *A. pinnata*, stress increased the contents of asparagine and glycine. In both species, the methionine biosynthesis was reduced. In terrestrial plants, the increased levels in some of these amino acids have previously been shown to be triggered by abiotic stress [77,78,79,80,81,82].

## 4. Conclusions

**Figure 5:**
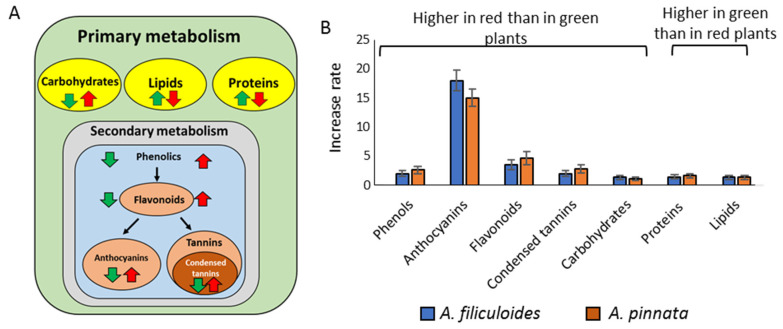
(**A**) Representation of differential accumulation of cellular primary (carbohydrates, lipids, and proteins) and secondary (phenolics, anthocyanins, flavonoids, and condensed tannins) metabolites in red (red arrow) and greed (green arrow) plants; (**B**) comparative increase rates (fold increase, *p* < 0.05) in production of metabolites between red and green Azolla plants.

Azolla is a novel sustainable feed supplement, producing compounds that can be broadly assessed under fully controlled indoor conditions. However, globally, a large majority of farmers are growing plants outdoors, and as a result, the plant’s nutritional value can be affected by environmental and nutrient conditions. The changes in key components, phenolics, proteins, carbohydrates, and proteins were summarized in Figure 5. Anthocyanins, flavonoids, and condensed tannins were observed to be the most induced nutrients in response to stresses. Increased levels of these molecules, decreased concentrations of lipids and proteins, along with changes in amino acid profiles could have a complex impact on the nutritional value of these plants.

## Figures and Tables

**Table 1:** Concentrations of nitrogen, total amino acids (AA) and proteins in *A. filiculoides* and *A. pinnata*.

	Total N mg/g DW	Total Protein *, mg/g DW	Total Protein **, mg/g DW	Total Protein ***, mg/g DW	Total AA, mg/g	N-Prot Factor
AFG	38.1 ± 2.5 ^#^	237.5 ± 22.2	174.42 ± 12.1	190 ± 9.1	205.3 ± 8.1 ^#^	5.40
AFR	26.4 ± 3.2 ^#^	165 ± 8.3	121.176 ± 10.1	132 ± 10.8	140.9 ± 7.4 ^#^	5.34
APG	46.0 ± 4.1 ^#^	287.5 ± 15.4	211.14 ± 14.4	230 ± 18.5	265.0 ± 10.4 ^#^	5.76
AFR	27.5 ± 2.3 ^#^	171.8 ± 10.0	126.225 ± 8.8	137.5 ± 6.6	134.0 ± 4.3 ^#^	4.87

AFG: *A. filiculoides*_Green; AFR: *A. filiculoides*_Red; APG: *A. pinnata*_Green; APR: *A. pinnata*_Red. Nitrogen-to-protein conversion factors: * 6.25 [11]; ** 4.59 [75]; *** 5.0 [76]. AA: amino acids. ^#^ Significance levels: *p* < 0.05.

**Table 2:** Amino acid concentrations in biomass of *A. filiculoides* and *A. pinnata* grown under normal and stress conditions.

AA	AA Content, % DW				AA Content, mg/g				AA Content, % in Total AA			
	AFG	AFR	APG	APR	AFG	AFR	APG	APR	AFG	AFR	APG	APR
Ala	0.46 ± 0.01	0.44 ± 0.1	0.62 ± 0.1	0.27 ± 0.01	4.6 ± 0.1	4.35 ± 0.1	6.22 ± 0.02	2.705 ± 0.1	2.26 ± 0.02	3.07 ± 0.02	2.32 ± 0.01	2.32 ± 0.02
Arg	2.3 ± 0.05	2.28 ± 0.07	3.1 ± 0.02	1.59 ± 0.08	22.97 ± 0.5	22.81 ± 0.65	31.03 ± 0.19	15.93 ± 0.8	11.21 ± 0.01	16.12 ± 0.02	11.55 ± 0.03	11.51 ± 0.1
Asp	2.08 ± 0.07	1.55 ± 0.1	2.47 ± 0.1	1.45 ± 0.06	20.8 ± 0.66	15.46 ± 0.47	24.5 ± 0.11	14.46 ± 0.6	10.15 ± 0.11	10.92 ± 0.12	9.19 ± 0.06	10.45 ± 0.02
Cys	ND	ND	ND	ND	ND	ND	ND	ND	ND	ND	ND	ND
Glu	4.51 ± 0.09	2.09 ± 0.06	6.94 ± 0.04	4.29 ± 0.02	45.1 ± 0.9	20.89 ± 0.64	69.37 ± 0.36	38.92 ± 2.1	22.00 ± 0.05	14.76 ± 0.10	25.83 ± 0.04	31.3 ± 0.16
Gly	1.27 ± 0.02	0.75 ± 0.02	1.56 ± 0.02	0.85 ± 0.03	12.67 ± 0.16	7.50 ± 0.20	15.55 ± 0.16	8.50 ± 0.3	6.18 ± 0.06	5.30 ± 0.06	5.79 ± 0.04	5.79 ± 0.02
His *	ND	ND	ND	ND	ND	ND	ND	ND	ND	ND	ND	ND
Ile *	1.06 ± 0.03	0.76 ± 0.02	1.29 ± 0.02	0.59 ± 0.02	10.57 ± 0.25	7.59 ± 0.21	12.89 ± 0.03	5.87 ± 0.27	5.16 ± 0.01	5.37 ± 0.02	4.80 ± 0.02	4.25 ± 0.02
Leu *	1.8 ± 0.03	1.34 ± 0.03	2.26 ± 0.02	1.31 ± 0.04	17.95 ± 0.32	13.35 ± 0.37	22.61 ± 0.017	11.5 ± 0.49	8.76 ± 0.04	9.43 ± 0.04	8.42 ± 0.03	7.99 ± 0.06
Lys *	1.43 ± 0.02	0.96 ± 0.03	1.64 ± 0.02	0.53 ± 0.03	14.34 ± 0.2	9.59 ± 0.32	16.38 ± 0.21	5.34 ± 0.32	7.00 ± 0.11	6.78 ± 0.13	6.10 ± 0.06	6.03 ± 0.1
Met *	1.22 ± 0.04	0.62 ± 0.02	1.58 ± 0.02	0.37 ± 0.01	12.25 ± 0.35	6.18 ± 0.20	15.75 ± 0.21	4.85 ± 0.19	5.96 ± 0.05	4.37 ± 0.03	5.87 ± 0.08	3.51 ± 0.08
Phe *	0.92 ± 0.02	0.62 ± 0.02	1.14 ± 0.00	0.54 ± 0.002	9.15 ± 0.22	6.24 ± 0.18	11.36 ± 0.03	5.35 ± 0.24	4.47 ± 0.01	4.41 ± 0.02	4.23 ± 0.02	3.87 ± 0.01
Pro	0.09 ± 0.02	0.06 ± 0.01	0.11 ± 0.02	0.06 ± 0.01	0.91 ± 0.16	0.61 ± 0.11	1.09 ± 0.18	ND	0.44 ± 0.07	0.43 ± 0.08	0.41 ± 0.07	ND
Ser	0.94 ± 0.02	0.65 ± 0.02	1.15 ± 0.01	0.6 ± 0.02	9.4 ± 0.16	6.49 ± 0.17	11.54 ± 0.08	6.05 ± 0.2	4.6 ± 0.03	4.59 ± 0.03	4.3 ± 0.02	4.37 ± 0.00
Thr *	0.9 ± 0.02	0.73 ± 0.02	1.05 ± 0.01	0.44 ± 0.02	9.01 ± 0.24	7.25 ± 0.20	10.52 ± 0.06	4.45 ± 0.22	4.40 ± 0.02	5.12 ± 0.02	3.92 ± 0.04	3.22 ± 0.01
Tyr	0.33 ± 0.01	0.33 ± 0.01	0.51 ± 0.00	0.12 ± 0.02	3.2 ± 0.05	3.29 ± 0.08	5.12 ± 0.04	1.17 ± 0.06	1.59 ± 0.02	2.33 ± 0.02	1.91 ± 0.01	1.91 ± 0.02
Val *	1.23 ± 0.03	0.93 ± 0.03	1.48 ± 0.00	0.72 ± 0.01	12.26 ± 0.34	9.35 ± 0.27	14.84 ± 0.04	7.19 ± 0.35	5.98 ± 0.03	6.60 ± 0.05	5.53 ± 0.03	5.20 ± 0.01
Total	20.5 ± 0.4	14.1 ± 0.2	26.5 ± 0.4	14.0 ± 0.5	205.3 ± 8.01	140.9 ± 7.4	265.9 ± 10.4	134 ± 4.3	100.1 ± 0.9	99.6 ± 0.8	100.1 ± 0.6	99.3 ± 0.1

* Essential amino acids; AFG: *A. filiculoides*_Green; AFR: *A. filiculoides*_Red; APG: *A. pinnata*_Green; APR: *A. pinnata*_Red. AA: amino acids. Significance levels: *p* < 0.05; ND-Not detected; Alanine (Ala); Arginine (Arg); Aspartic acid (Asp); Cysteine (Cys); Glutamicacid (Glu); Glycine (Gly); Histidine (His); Isoleucine (Ile); Leucine (Leu); Lysine (Lys); Methionine (Met); Phenylalanine (Phe); Proline (Pro); Serine (Ser); Threonine (Thr); Tyrosine (Tyr); Valine (Val).

## Supplementary Materials

The following are available online at https://www.mdpi.com/2079-7737/9/10/342/s1, Figure S1: (A) *A. filiculoides* green (a) and red (b) grown outdoors. (B) *A. pinnata* green (a) and red (b) grown outdoors. Figure S2. (A) Longitudinal sections of *A. filiculoides* plants. (B) *Nostoc azollae* in leaf cavities shown by arrow.
