# Supplementary material for: Differential Production of Phenolics, Lipids, Carbohydrates and Proteins in Stressed and Unstressed Aquatic Plants, Azolla filiculoides and Azolla pinnata
Source: Biology (Basel). 2020 Oct 19;9(10):342. doi: 10.3390/biology9100342 (PMC7603371; doi:10.3390/biology9100342)
Supplement: Supplementary file 1 [file biology-09-00342-s001.pdf]

RMIT Classification: Trusted

A

B

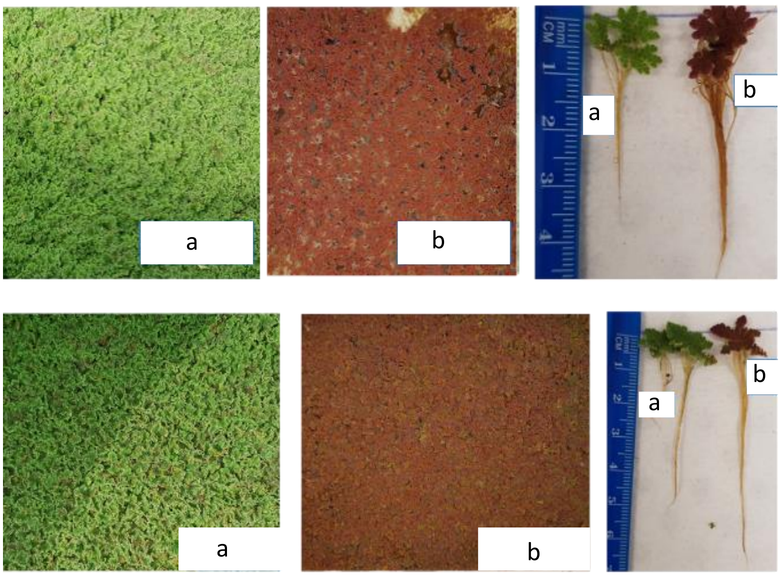

Fig S1: (A) *A. filiculoides* green (a) and red (b) grown outdoors. (B) *A. pinnata* green (a) and red (b) grown outdoors.

RMIT Classification: Trusted

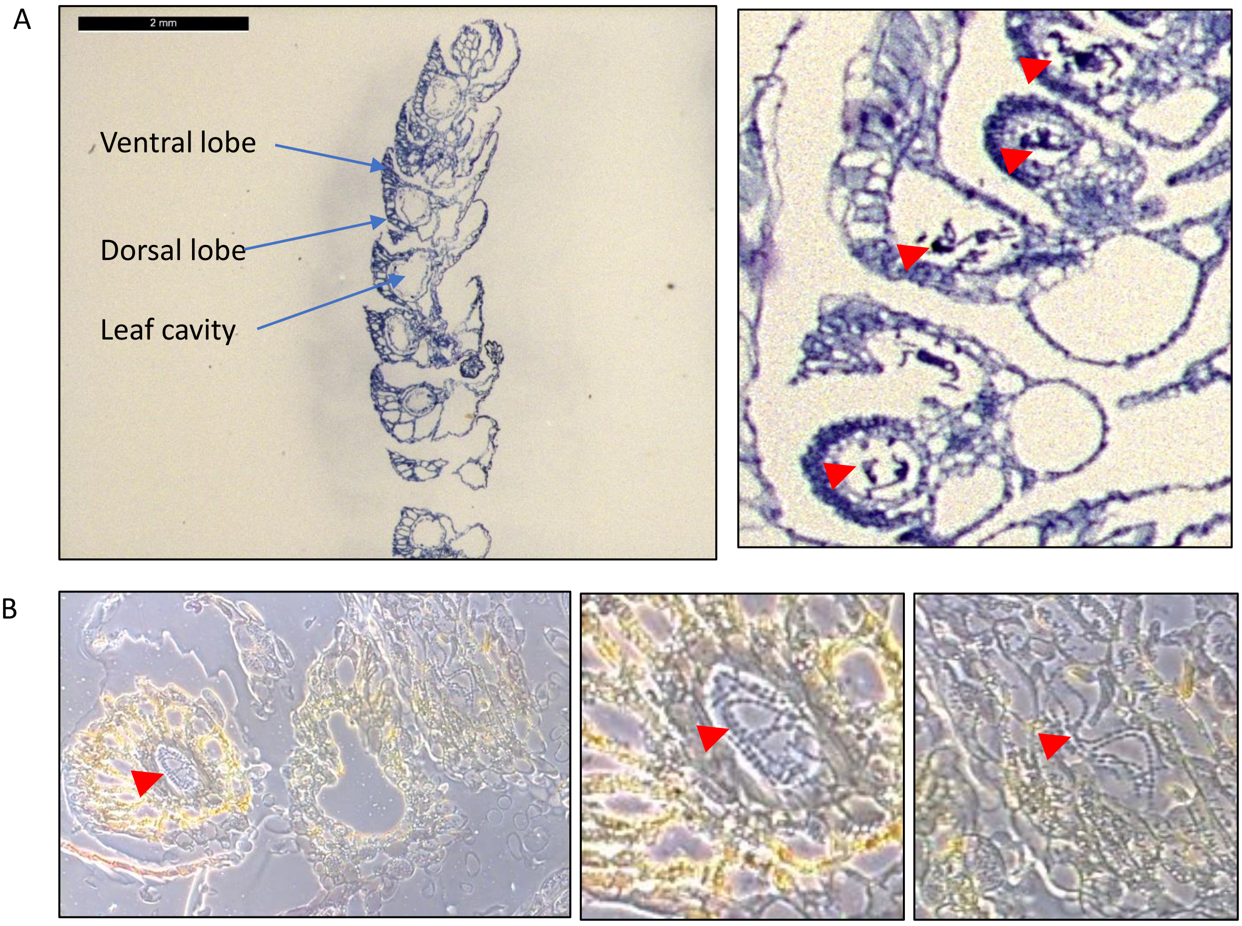

Figure S2. (A) Longitudinal sections of *A. filiculoides* plants. (B) *Nostoc azollae* in leaf cavities shown by arrow
